# An Online Acceptance and Commitment Therapy Session to help People Manage Stress from their Dementia Diagnosis

**DOI:** 10.1093/geroni/igaf122.2595

**Published:** 2025-12-31

**Authors:** Gavin Green, Heather Kelley, Ty Aller, Carter Davis, Michael Levin, Elizabeth Fauth

**Affiliations:** Utah State University, Logan, Utah, United States; Utah State University, Logan, Utah, United States; Utah State University, Logan, Utah, United States; VA Palo Alto Health Care System, Palo Alto, California, United States; Utah State University, Logan, Utah, United States; Utah State University, Logan, Utah, United States

## Abstract

**Background:**

A dementia diagnosis means that a person faces forthcoming deterioration in function and eventually, death, thus these diagnoses are accompanied by significant psychological distress. While some CBT-based interventions have been found preliminarily effective in improving well-being for this population, there are significant barriers (e.g. time and cost). This study examines the Compassion Compass, a single-session, online Acceptance and Commitment Therapy (ACT) program tailored to support individuals recently diagnosed with dementia.

**Methods:**

Eighteen participants (mean age = 66.1, SD = 11.9), diagnosed with dementia within the past year, completed Compassion Compass. Preliminary efficacy was assessed using quantitative and qualitative measures. Quantitative measures assessed changes in psychological distress and life satisfaction, while in-depth interviews provided qualitative insights into the intervention’s perceived impact on participants’ daily lives and well-being.

**Results:**

Quantitative findings, limited by the sample size, showed no statistically significant improvement in life satisfaction, but yielded a marginal reduction in psychological distress. Qualitative data highlighted meaningful benefits. Thematic analysis revealed increased emotional awareness, self-compassion, and values-based engagement. Participants reported feeling better able to process their diagnosis, reframe their perspective, and reconnect with meaningful activities.

**Conclusion:**

Though the study was under powered to examine efficacy quantitatively, its qualitative findings suggest that the Compassion Compass program was well-received by participants and helped support their well-being, making it a promising, low time and cost option that warrants further research. Future research will refine the intervention and explore its potential within caregiver care-recipient dyads to further improve well-being for individuals with dementia.

